# Prevention and management of radiodermatitis in patients with brachytherapy for gynecologic neoplasms summary of the evidence

**DOI:** 10.3389/fonc.2025.1645101

**Published:** 2025-08-08

**Authors:** Zhanxin Fan, Hongxia Liang, Xin Wei, Bowen Cui, Lin Wang

**Affiliations:** ^1^ Department of Abdominal Radiation Oncology, Zhongshan People’s Hospital, Zhongshan, China; ^2^ College of Nursing and Health, Henan University, Kaifeng, China; ^3^ Headquarters of Cancer Branch, Zhongshan People’s Hospital, Zhongshan, China

**Keywords:** gynecologic neoplasms, brachytherapy, radiodermatitis, evidence summary, evidence-based nursing

## Abstract

**Objective:**

This study aims to systematically retrieve, evaluate, and summarize the best evidence regarding the prevention and management of radiation dermatitis in patients undergoing endoluminal radiotherapy for gynecologic neoplasms. The goal is to provide an evidence-based foundation for developing personalized skin management programs.

**Methods:**

We conducted a comprehensive search of domestic and international databases, guideline networks, and the websites of relevant professional associations to identify all evidence related to the prevention and management of radiation dermatitis in patients receiving endoluminal radiotherapy for gynecologic neoplasms. The types of literature include guidelines, evidence summaries, systematic reviews, expert consensus, clinical decision-making, and randomized controlled trials. The search period spanned from database inception to September 2024. In addition, we performed hand-searching of key journals and tracked gray literature (including conference abstracts and unpublishe doi: d reports).

**Results:**

The review included 14 articles, including 1 clinical decision-making paper, 4 guideline articles, 3 systematic reviews, 3 evidence summary papers, 1 expert consensus document, and 2 randomized controlled trials. We summarized 27 pieces of evidence categorized into 5 themes: assessment and evaluation, health education, general nursing measures, symptom management, and continuity of care.

**Conclusions:**

This study consolidates the most effective evidence for managing radiation dermatitis in patients receiving three-dimensional brachytherapy for gynecologic tumors. The findings can serve as a valuable resource for minimizing skin damage and enhancing patients’ quality of life.

**Clinical Trial Registration:**

http://ebn.nursing.fudan.edu.cn/home, identifier ES20256976.

## 1 Introduction

In recent years, the incidence of gynecologic neoplasms in China has been increasing annually, with approximately 290,000 new cases of gynecologic cancer and about 100,000 deaths in 2022 (1). Among these, the three most commonly diagnosed gynecologic neoplasms are cervical cancer, endometrial cancer, and ovarian cancer (2). Most patients are diagnosed at middle to advanced stages, and chemoradiotherapy is predominantly used in clinical practice (3).

The traditional standard treatment strategy for locally advanced gynecologic tumors involves external beam radiotherapy (EBRT) combined with concurrent chemotherapy, followed by intracavitary brachytherapy (ICBT) (4). Endovascular brachytherapy, also referred to as intraluminal post-loading radiotherapy, is typically administered at a total dose of 24 Gy over six sessions, once a week (5). According to the radiotherapy protocol, patients are usually admitted to the hospital the day before each treatment and can be discharged without discomfort on the same day as the procedure. This approach has become a mainstream method in radiotherapy technology and is widely utilized in clinical settings (6). However, due to the combination of these two radiotherapy modalities and the location of treatment—primarily the perineum, which has a high density of nerve endings—radiation dermatitis (RD) is a common complication (7). RD typically presents as exudation and edema in the early stages, progressing to erosion, secondary infection, hemorrhage, and the eventual formation of ulcers in later stages (8). The study indicates that grade 1 and 2 radiation dermatitis are relatively common among gynecologic cancer patients undergoing radiotherapy, with incidence rates ranging from 10% to 50% in cervical and endometrial cancer patients. However, severe radiation (grade 3 or higher) dermatitis is relatively rare, generally occurring in 1% to 5% of cases (9–12). It significantly impacts patients’ quality of life and treatment adherence. However, current research mainly provides general skin care advice and lacks a systematic review of nursing strategies specific to this group. Because gynecological tumors involve unique anatomy and radiation sites, their dermatitis has different characteristics that are not well understood or managed with existing protocols. Furthermore, there are no widely accepted, evidence-based nursing guidelines tailored to these patients, including the best topical medications, dressings, and nursing interventions. Most current practices are based on limited or isolated evidence, which makes it harder to improve care and reduce skin problems effectively.

This study aims to fill these gaps by reviewing the existing evidence to identify effective prevention and treatment strategies for radiation dermatitis in gynecological patients. The ultimate goal is to establish standardized nursing protocols to reduce the incidence of radiation dermatitis, improve patient outcomes, and enhance quality of life. This study has been registered on the website of the Center for Evidence-Based Nursing at Fudan University (registration number: ES20256976).

## 2 Information and methods

### 2.1 Establishment of evidence-based issues

Based on the “PIPOST” problem model developed by the Center for Evidence-Based Nursing at Fudan University, we constructed the following evidence-based question (13). Population (P): Patients with gynecologic tumors receiving intracavitary brachytherapy radiation therapy. Intervention (I): Strategies for the prevention and management of radiation dermatitis. Professional (P): Medical staff, patients, and their families. Outcome (O): The occurrence of radiation dermatitis. Setting (S): Gynecologic oncology ward, outpatient clinic, and home care settings. Type of Evidence (T): Guidelines, evidence summaries, expert consensus, clinical decision-making resources, clinical practice documents, systematic reviews, and high-quality randomized controlled trials.

### 2.2 Literature search strategy

**Figure 1:**
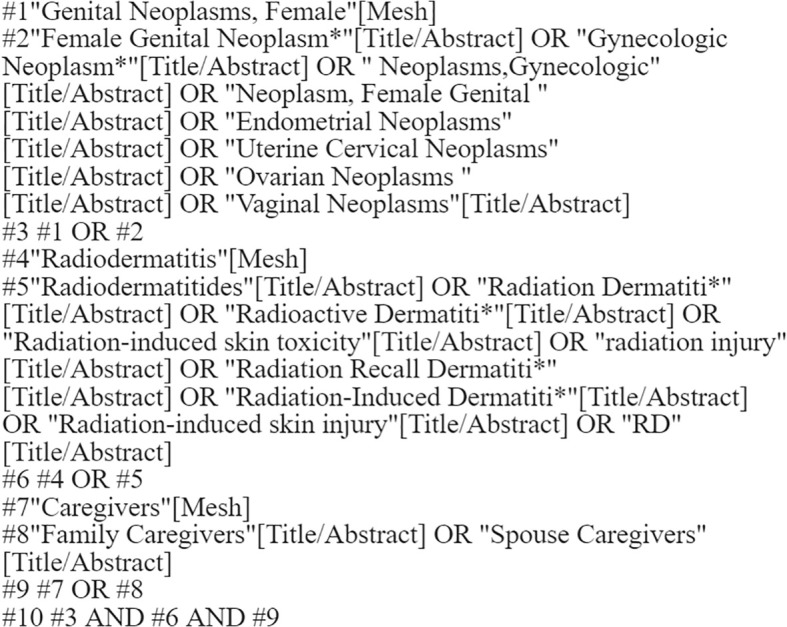
PubMed search strategy.

Following the top-down search principle of the evidence-based “6S” pyramid model, we conducted a comprehensive search using various resources (14). We searched BMJ Best Practice and UpToDate as key evidence-based resources. The evidence-based databases included the Cochrane Library and the Joanna Briggs Institute (JBI), while comprehensive databases comprised CINAHL, Web of Science (WOS), PubMed, Wiley Online, and Embase. Additionally, we explored websites such as the Guidelines International Network (GIN), the American Guideline Network, the Scottish Intercollegiate Guidelines Network, and professional association sites like the Registered Nurses Association of Ontario (RNAO). We also reviewed several Chinese databases, including the Wanfang Database, China National Knowledge Infrastructure (CNKI), the Chinese Biology Medicine (CBM) Database, and the VIP Chinese Science and Technology Journal Database (VIP). The search utilized the following subject terms and free-text keywords: “genital neoplasms, female/female genital neoplasms/gynecologic neoplasms/neoplasms, gynecologic/neoplasm, female genital”, “Endometrial Neoplasms/Uterine Cervical Neoplasms/Ovarian Neoplasms/Vaginal Neoplasms”, “radiodermatitis/radiation dermatitis/radioactive dermatitis/radiation-induced skin toxicity/radiation injury/radiation recall dermatitis/radiation-induced dermatitis/radiation-induced skin injury/RD” and “caregivers/family caregivers/spouse caregivers”. The search timeframe extended from the establishment of each database until September 2024. In addition, we performed hand-searching of key journals and tracked gray literature (including conference abstracts and unpublished reports). The PubMed search strategy, shown in **Figure 1**, was systematically constructed using the following approach:

1. Population component (#1-#3): Combines MeSH terms and free-text terms for gynecological cancers (e.g., cervical, ovarian, endometrial, vaginal neoplasms).
2. Outcome component (#4-#6): Incorporated both controlled vocabulary (‘Radiodermatitis’ MeSH) and comprehensive free-text terms for radiation-induced skin toxicity.
3. Contextual component (#7-#9): Included caregiver-related terms to capture psychosocial aspects.
4. Final combination (#10): Intersected all concept groups using Boolean AND operator.

The strategy was optimized through iterative testing to balance sensitivity (recall) and specificity (precision).

### 2.3 Literature inclusion and exclusion criteria

Inclusion Criteria: The study must focus on management and prevention strategies for radiation dermatitis. Eligible study types include: Guidelines, Expert consensus, Evidence summaries, Clinical decision-making resources, Clinical practice documents, Systematic Reviews, and randomized controlled trials, Publications must be available in both Chinese and English. Exclusion Criteria: Original studies that focus solely on pharmacological interventions, Articles lacking complete information, Publications without full-text access, Duplicate publications, Studies that failed the literature quality assessment, low correlation studies (e.g., those with study populations differing from the target population in PIPOST or failing to report prespecified outcome measures), investigate articles (e.g., uncontrolled observational designs, small-scale cross-sectional studies, or non-standardized case series).

### 2.4 Literature quality evaluation criteria

The guideline evaluation process was carried out independently by four experts using the Appraisal of Guidelines for Research and Evaluation (AGREE II) system (15). The assessment included 23 items across six domains: scope and purpose, participants, rigor of formulation, clarity, applicability, and editorial independence. Each item was rated on a 7-point scale, where 7 signifies full compliance and 1 indicates non-compliance.

Two researchers with expertise in evidence-based knowledge evaluated other types of literature. The literature that met the inclusion criteria was compiled, and its quality was assessed independently. The quality of included systematic reviews and expert consensus documents was evaluated using the JBI Evaluation Criteria for Evidence-Based Health Care Centers (2016 edition) (13). Randomized controlled trials were assessed using the Cochrane Risk of Bias Tool (16). The quality assessment of evidence summarization and clinical decision-making traces back to the original literature, followed by evaluating its quality using appropriate assessment standards according to the type of literature.

### 2.5 Evidence extraction, synthesis, and evaluation

In cases where contradictory conclusions arise from different pieces of literature, this study will adhere to the principles of evidence-based practice, prioritizing high-quality evidence, the most recently published authoritative literature, and domestic guidelines. The integration of results will be reviewed by a third researcher.

The JBI Evidence Pre-Grading and Evidence Recommendation Level System (2014 Edition) was employed to classify the original literature identified in the final included evidence (17). This classification system ranks evidence from levels 1 to 5, with level 1 being the highest quality and level 5 the lowest. The recommendation grades were determined based on the FAME attributes of the evidence (Feasibility, Appropriateness, Meaningfulness, and Effectiveness), with Grade A indicating strong recommendation and Grade B indicating weak recommendation.

## 3 Results

### 3.1 General characteristics of the included literature

This study retrieved 748 relevant articles from database searches. Additionally, by reviewing related meta-analyses, reviews, and the reference lists of included articles, relevant studies were identified through hand-searching. Finally, five more articles were obtained through manual searching in the library, bringing the total number of articles to 753. After excluding duplicates, articles that did not meet the topic criteria, and other irrelevant literature, an initial total of 33 eligible articles were identified. Following quality assessment of these articles, 26 articles with poor quality were excluded, resulting in a final inclusion of 14 articles.

**Figure 2:**
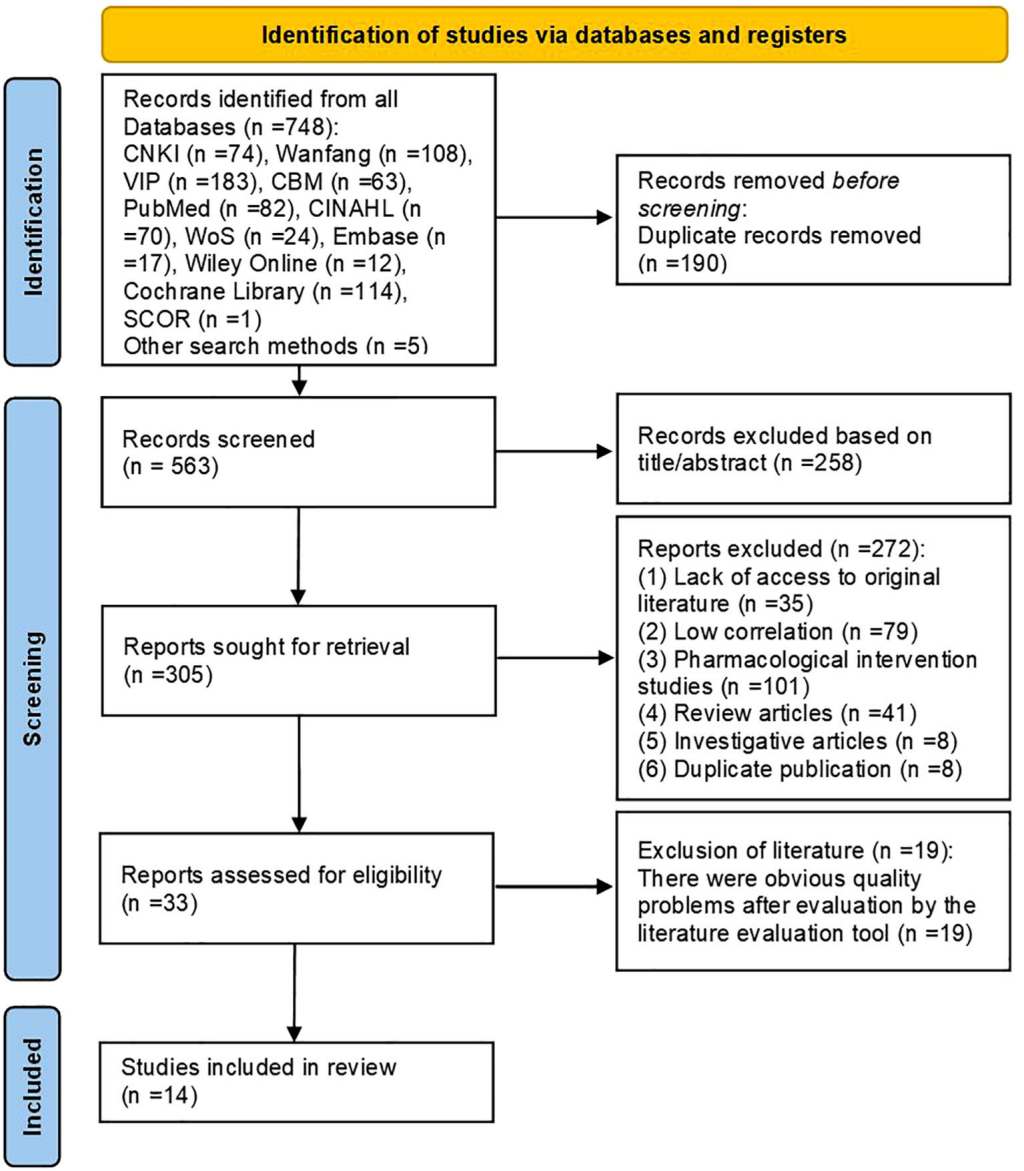
Flow diagram of literature search.

**Figure 3:**
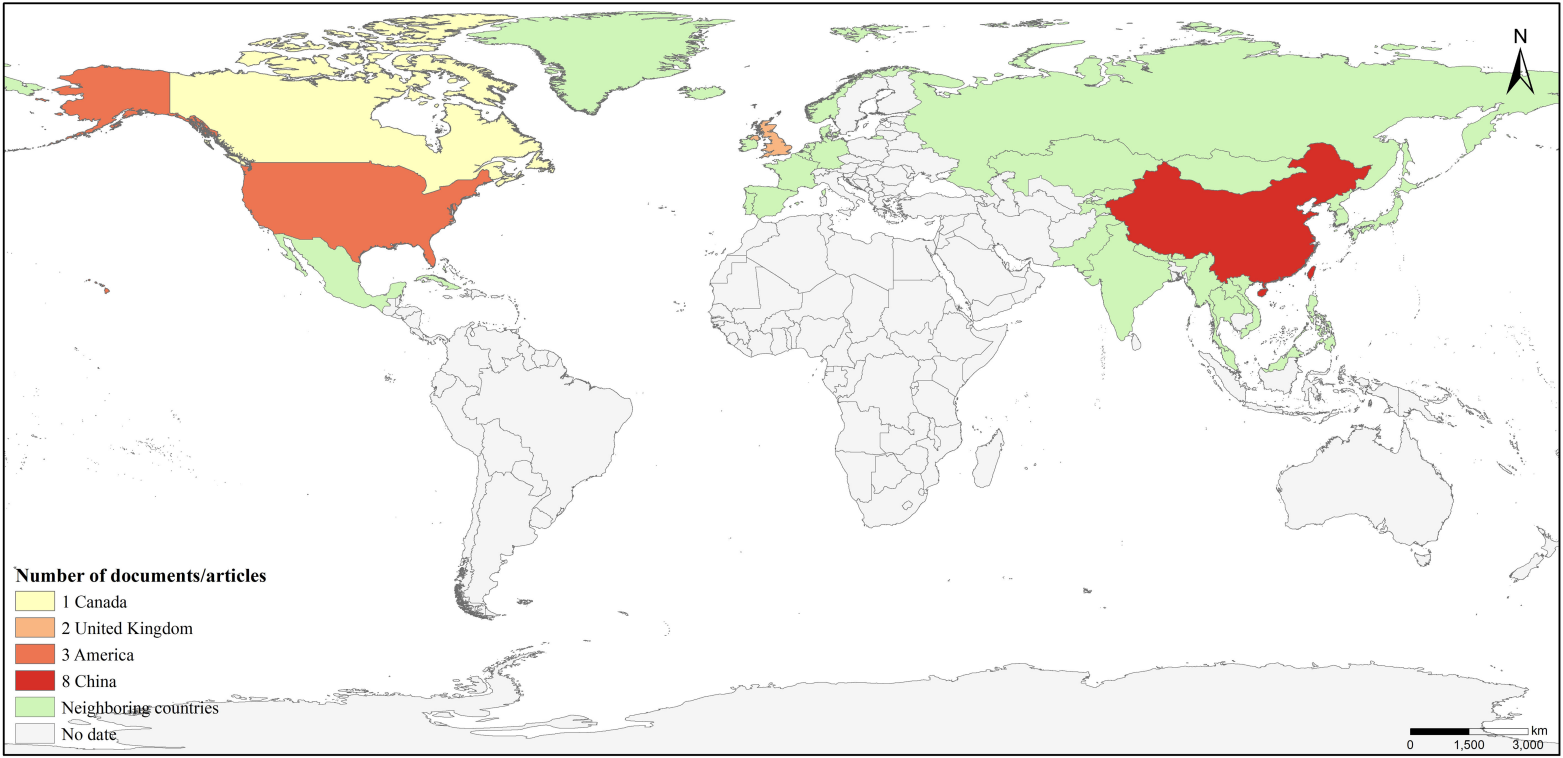
Map figure.

This included: 1 clinical decision (18), 4 guideline articles (19–22), 3 systematic reviews (23–25), 3 evidence summaries (26–28), 1 expert consensus (29) and 2 randomized controlled studies (30, 31). The flow chart of the literature retrieval process is presented in **Figure 2**, accompanied by a map (**Figure 3**) illustrating the global distribution. Additionally, the overall characteristics of the included literature are summarized in **Table 1**.

**Table 1:** Basic characteristics of included studies (n=14).

Producer/Author	Publication Year	Literature Source	Type of Evidence	Language	Literature Topics
Yan et al. (31).	2018	CNKI	RCT	Chinese	Application of predictive care in patients with endoluminal posterior radiotherapy for advanced cervical cancer
Mai et al. (30).	2013	CNKI	RCT	Chinese	Application of nursing intervention in intensity-modulated post-loading radiotherapy for recurrent cervical cancer
Tracy Gosselin et al. (19).	2020	PubMed	Guideline	English	Development of a management approach to radiation dermatitis
Wong et al. (20).	2013	CINAHL	Guideline	English	Summary of various approaches to the treatment of radiation dermatitis in cancer patients on an evidence-based basis
ScoR Radiotherapy Working Group (21).	2021	ScoR	Guideline	English	Prescribe the management of radiation dermatitis
Harries et al. (22).	2017	PubMed	Guideline	English	Clinical guidelines for acute radiotherapy-induced skin reactions in cancer patients
Wang et al. (23).	2020	CNKI	Systematic Review	Chinese	Systematic review of the effect of skin cleansing on radiation dermatitis in cancer patients
Liu et al. (24).	2023	VIP	Systematic Review	Chinese	Re-evaluation of systematic reviews related to radiation dermatitis prophylaxis in cancer patients
Cao et al. (25).	2024	Embase	Systematic Review	English	A systematic review of the effects of radiation dermatitis interventions
Backler et al. (26).	2020	PubMed	Summary of Evidence	English	Clinical summary of guidelines for radiation dermatitis associated with cancer treatment
Wang et al. (27).	2021	VIP	Summary of Evidence	Chinese	Summary of the evidence for the prevention and management of radiation dermatitis
Chen et al. (28).	2021	VIP	Summary of Evidence	Chinese	Review of guidelines for the prevention and management of radiation dermatitis, summarizing the best evidence
Wolf et al. (18).	2023	Up to Date	Clinical Decision-Making	English	Summary of radiation dermatitis prevention and treatment
Burn Trauma Branch of Chinese Geriatrics Society et al. (29).	2024	WanFang	Expert Consensus	Chinese	Expert consensus on the diagnosis and treatment of radiation skin injury

### 3.2 Quality evaluation results of the included literature

#### 3.2.1 Quality evaluation results of the guidelines

A total of four guidelines were included in this study (19–22), of which, three guidelines received standardized scores of ≥ 60 percent across six domains and were rated as A, while the remaining guideline was rated as B, as detailed in **Table 2**.

**Table 2:** Results of the quality evaluation of the guidelines.

Inclusion Guidelines	Standardized scores in various domains (%)						≥ 60% Field Number(n)	≥30% Field Number (n)	Recommendation level
	Scope and Purpose	Involved Personnel	The rigor of the Guidelines	Clarity of Guidelines	Applicability	Editorial independence			
Gosselin et al. (19). Wong et al. (20). Harries et al. (22). ScoR Radiotherapy Working Group (21).	83.3 88.9 94.4 83.3	88.9 88.9 90.7 100	91.7 75 96.5 95.8	83.3 83.3 91.7 94.4	79.2 58.3 98.6 83.3	100 100 94.4 100	6 5 6 6	0 0 0 0	A B A A

#### 3.2.2 Quality evaluation results of the systematic review

A total of three relevant systematic reviews were included in this study (23–25). The evaluation results were as follows:

Review (23): Item 9 regarding the assessment of publication bias is “unclear,” while all other items are “yes”.
Review (24): The evaluation result for “whether error minimization was sufficiently attempted during data extraction (Item 8)” was “unclear”.
Review (25): All items were rated “yes”.

The full evaluation results are presented in **Table 3**.

**Table 3:** Quality evaluation results of the systematic review.

Systematic Reviews Inclusion and Evaluation	Evaluation items											Inclusion (Yes/No)
	①	②	③	④	⑤	⑥	⑦	⑧	⑨	⑩	⑪	
Wang et al. (23). Liu et al. (24). Cao et al. (25).	yes yes yes	yes yes yes	yes yes yes	yes yes yes	yes yes yes	yes yes yes	yes yes yes	yes unclear yes	unclear yes yes	yes yes yes	yes yes yes	yes yes yes

① Is the evidence-based question raised clear and explicit? ② Is the inclusion criteria for literature appropriate for this evidence-based question? ③ Is the retrieval strategy appropriate? ④ Is the search database or resources sufficient? ⑤ Is the literature quality evaluation standard used appropriately? ⑥ Are there 2 or more evaluators independently completing quality evaluations? ⑦ Are certain measures taken to reduce errors when extracting data? ⑧ Is the method of merging research appropriate? ⑨ Has the possibility of publication bias been evaluated? ⑩ Are the policy or practice recommendations based on the results of a systematic evaluation? ⑪ Is the proposed further research direction appropriate?

#### 3.2.3 Quality evaluation results of clinical decision-making and evidence summarization

In this study, one clinical decision and three evidence summaries were included (18, 26–28). The original literature that retrospectively aligned with this study included two guidelines (20, 21), both of which were accepted following the quality evaluation of the aforementioned literature. In one systematic review (32), the results were “unclear” in the “appropriateness of the quality standards used”, “no” in the “whether measures were used to reduce errors when extracting data” and “whether possible publication bias was assessed”, and “yes” for all other items, not included; A review was evaluated according to expert opinions (33), and the evaluation results were “unclear” in the items “whether the views are derived from influential experts in the field” and “whether there are any inconsistencies between the opinions presented and previous literature”, and “yes” in all other items, not included.

#### 3.2.4 Quality evaluation results of expert consensus

In this study, one expert consensus was included (29). The evaluation results for all items were rated as “yes”.

#### 3.2.5 Quality evaluation results of randomized controlled studies

In this review, two randomized controlled trials were included (30, 31). Two studies were included in the “yes” with the exception of “allocation concealment”, “blinding of investigators and participants”, and “blinding of outcome assessors” as “unclear”.

### 3.3 Summary of evidence

A total of twenty-seven pieces of relevant evidence were preliminarily extracted. The contents related to the same topics were classified and summarized through group analysis, comparison, and discussion. This process resulted in the formation of five main themes:

Record and Evaluation.
Health Education.
General Nursing Measures.
Symptom Management.
Continuous Care.

These themes are presented in **Table 4**.

**Table 4:** Literature extraction.

Evidence items	Evidence content	Level of evidence	Recommended level
Record and Evaluation Health Education General Nursing Measures Symptom Management Continuous Care	1. Use RTOG grading criteria for skin assessment (19, 23, 25, 27, 28). 2. Evaluations should be conducted by trained personnel, with regular assessments of differences between clinicians, radiation therapy technologists, and oncology nurses (21, 28). 3. A baseline skin assessment should precede radiotherapy, and a skin file should be established to monitor the patient’s condition (28). 4. Dynamic assessments during treatment: weekly for grades 0–2 and daily for grades 3–4 and significant symptoms. Increase assessment frequency for high-risk patients to at least twice a week (21, 28). 5. The assessment encompasses the hygiene and cleanliness status of the perineal skin, the risk factors contributing to the development of local RD, as well as the skin care products currently being utilized by the patient (21, 28). 6. Document patient acceptability, satisfaction, and adherence to skincare recommendations (28). 7. Patients should be educated about skin management (26, 30, 31); 8. In light of the patient’s specific circumstances, select the most suitable educational approach (30). 9. Tabular materials that are at a sixth-grade reading level are the materials of choice for patient education (20, 28). 10. The content of health education encompasses patiently elaborating on the knowledge of intraluminal brachytherapy and radiation dermatitis to patients, as well as providing standardized skin care education (30). It also includes introducing the brands and features of the recommended products (20). Additionally, patients should be instructed to conduct self-monitoring of their skin conditions and to actively seek consultations with their healthcare providers (21). 11. Opt for wearing loose, soft garments made of breathable fabrics, such as cotton. Try to refrain from using sanitary napkins or pads as much as possible. For infants, use cotton diapers instead. Change undergarments frequently and make every effort to avoid rubbing the skin in the irradiated area (30). Before radiotherapy, patients should defecate to empty their bowels. Ensure that the radiation area remains clean and dry, and avoid exposing it directly to sunlight (18, 20, 27, 30, 31). 12. It is advisable to avoid using tapes and adhesives, as well as refrain from applying products like depilatory creams within the treatment area. Additionally, the application of ice or heat directly on the skin at the radiotherapy site is strictly prohibited (21, 28). Moreover, swimming is not allowed during radiation therapy (19, 27). 13. Patients are strongly advised against self-disposing of the blisters that appear at the radiotherapy site (27). 14. Caregivers should guide patients to persistently uphold their hygiene habits. During the treatment period, caregivers should also respect patients’ personal preferences and lifestyle patterns (18, 28). The skin can be cleansed using water and/or non-irritating soap. After that, the affected area should be gently patted dry (20, 21, 24, 25). Moreover, taking a bath is prohibited (19, 27). 15. Topical antibiotics should be avoided unless infection is confirmed (19, 28). 16. Aloe vera is not recommended (18, 19, 25, 27). 17. Inform patients that moisturizers are a standard part of skincare and can help prevent radiation dermatitis (19, 28). Unscented, lanolin-free moisturizers and antiperspirants can be applied after radiotherapy; however, they should be discontinued if the skin is broken. It’s preferable to use a moisturizer that does not contain sodium laurate (21). Patients should also avoid using topical moisturizers, gels, lotions, or dressings two hours radiotherapy (18). 18. For patients at risk of acute radiation dermatitis following radiation therapy, prophylactic use of radiation protection ointments or sprays is recommended. Patients should avoid using alcohol, iodophors, and other disinfectants on irradiated skin (20). 19. Do not scratch the skin in the treated area, and be sure to trim your nails weekly (27). 20. Patients are encouraged to monitor their skin conditions for signs of radiation dermatitis, such as increased sensitivity, pigmentation changes, and other skin reactions. These reactions may reach their peak approximately 10 to 14 days after the last treatment (21). 21. Ensure that the care team is informed if the patient requires ongoing wound management (21). 22. Patients are advised to clean wounds once or twice a day using water at a temperature of 36-40°C. They should avoid prolonged immersion of the wound during radiotherapy (29). 23. Provide clear explanations, encouragement, support, and guidance to help patients address their condition effectively. This will help them build confidence in overcoming the disease and foster a proactive attitude toward treatment compliance (31). 24. Class I radiation dermatitis, which does not require special management, can be prophylactically treated with topical steroids (e.g., mometasone, betamethasone) to help reduce discomfort and itching (18, 24, 27, 29). If the patient experiences itching at the irradiated site, the itchy area can be gently patted with the hand, and the affected skin should be cleaned with warm water (22). 25. For grade II or III radiation dermatitis, appropriate dressings (e.g., silver ion dressings or epidermal growth factor dressings) may be used to reduce the risk of further trauma and infection (18, 20, 22, 28). 26. For grade IV radiation dermatitis, the affected area may be washed with normal saline. Antibiotics should be administered as prescribed for infected wounds or those at risk of infection. Additionally, radiation therapy should be discontinued if necessary (18). 27. One day before discharge, provide patients with comprehensive discharge instructions. Emphasize the importance of maintaining skin care after discharge, observing for long-term complications, and attending regular follow-up appointments (30).	Level5 Level5 Level5 Level5 Level5 Level5 Level1 Level1 Level5 Level1 Level5 Level1 Level5 Level2 Level1 Level1 Level5 Level1 Level1 Level1 Level5 Level5 Level1 Level1 Level1 Level1 Level1	A B B A B B A B B A B A B A B A B A B A B B A B B B B

## 4 Discussion

### 4.1 Skin assessment and its documentation serve as the cornerstone for the prevention of radiation dermatitis

Currently, the most commonly used tool for clinical assessment of radiation dermatitis is the RTOG (Radiation Therapy Oncology Group) grading system (32). When using this tool to assess patients, the evaluation should be conducted by personnel who have received training in its use. Additionally, regular assessments should be performed to compare the differences in evaluations among clinicians, radiotherapy technicians, and radiotherapy nurses, ensuring the accuracy and consistency of radiation dermatitis assessments (21, 28). And it is necessary to dynamically assess and record the patient’s skin condition and symptoms that occur during radiotherapy (21, 22, 28). This allows for timely intervention. The assessment should include the condition of the perineal skin, hygiene and cleanliness, risk factors for the development of local radiation dermatitis, and the skin products currently used by the patient (21, 28). The results indicate that these measures can detect skin issues at an early stage and effectively reduce the incidence of radiation dermatitis (33, 34).

### 4.2 The importance of health education for patients undergoing postoperative intracavitary radiotherapy

Implantation-based intracavitary radiotherapy is usually initiated when external beam radiation therapy is completed or nearly completed, and the entire radiotherapy process lasts about two months (35). Unlike external beam radiation therapy, patients usually undergo only 1–2 sessions of radiotherapy per week, and they can be discharged after each session if they experience no unusual discomfort (36). Before the patient’s next admission for radiotherapy, medical staff are unable to monitor the patient’s skin changes during this period. Therefore, skin management and health education should be provided to the patient after the first intracavitary radiotherapy and before discharge (26, 30, 31). The content of health education includes patiently explaining relevant knowledge about radiotherapy and radiation dermatitis to the patient, providing standardized skin care education (30); recommending specific brands and images of products (20); guiding patients to self-monitor their skin and actively consult and discuss with healthcare professionals (21). The main approaches include oral guidance, written materials, and multimedia promotion, which significantly improve patients’ self-care ability and awareness of skin care, thereby reducing the occurrence of radiation dermatitis (30, 31). During the literature review, it was found that there is currently a lack of research on education and support strategies targeting patients’ family members. This gap limits the potential of family involvement in patient care and also suggests that future research should focus on developing and evaluating educational and support programs for family members to strengthen their role in preventing radiation dermatitis and to improve patient compliance and care outcomes.

### 4.3 Skin protection during radiotherapy is key to preventing radiation dermatitis

Daily skin care is crucial for preventing radiation dermatitis in patients undergoing radiotherapy as well as for promoting healing after its occurrence. Studies have shown that providing anticipatory guidance and care during the patient’s radiotherapy can effectively reduce the incidence of radiation dermatitis (37, 38). 20 pointed out that patients undergoing radiotherapy can use plain water and/or non-irritating, non-alkaline soap to wash their skin, which should become a routine clinical care measure (20). Several randomized trials involving breast cancer and head and neck cancer patients, as well as a meta-analysis, have evaluated the benefits of routine cleansing during treatment in preventing severe radiation dermatitis (39–42), but bath soaking should be avoided. In addition, non-irritating and highly moisturizing care products can be used, while avoiding irritant medications such as alcohol and povidone-iodine (19, 20). These measures help alleviate adverse reactions such as skin dryness and swelling. However, it is recommended that patients do not apply topical moisturizers, gels, lotions, or dressings within two hours before radiotherapy to prevent a bolus effect, which can increase the radiation dose to the epidermis (18, 43). At the same time, professional nursing guidance should avoid any measures that could cause local pressure or friction, thereby effectively controlling skin damage caused by radiation (30). In summary, reasonably preventing the bolus effect through scientific skin care measures is of great significance for improving treatment compliance and the quality of life in patients undergoing intracavitary postoperative radiotherapy.

### 4.4 The use of medications during radiotherapy is a key focus in managing radiation dermatitis

Compared to radiotherapy for head and neck cancer and breast cancer, cervical cancer radiation dermatitis receives less attention, and there is even a lack of relevant research on radiation dermatitis in patients undergoing three-dimensional brachytherapy (44). The irradiated area is located in the perineal region, where the skin is moist, wrinkled, and involves patient privacy. The characteristic features of radiation dermatitis in this area include the formation of papules and rashes, making treatment much more challenging than other forms of radiation dermatitis (7). Previous studies have used treatments such as Bao-Shi-Jie, Kangfu-Xin liquid, Fufang Tongye Burn Oil, and Cu Yu-Ling ointment for cervical cancer radiation dermatitis (45–47), with some reports indicating moderate effectiveness. However, these treatments have not yet been widely adopted. A clinical decision guideline recommends the use of corticosteroids (18), which have been shown to effectively reduce itching caused by radiation dermatitis. Additionally, a meta-analysis indicated that multiple randomized controlled trials confirmed that topical corticosteroids can decrease the risk of radiation dermatitis in breast cancer patients (48). In a randomized trial for the treatment of radiation dermatitis in breast cancer, sulfonamide silver demonstrated good anti-inflammatory effects and is often used to treat radiation dermatitis (49). Moreover, certain dressings, such as silicone gel dressings and silver-nylon dressings, have also been shown to be effective in treating Grade II or Grade III radiation dermatitis (50, 51). However, these medications and dressings are primarily used in breast cancer and head and neck cancer patients, and their applicability to cervical cancer radiotherapy patients has not yet been established. Therefore, regarding radiation dermatitis in patients with intracavitary postoperative radiotherapy for cervical cancer, more clinical research and trials are needed to validate the efficacy and safety of existing treatment options. Additionally, it is important to explore preventive and therapeutic methods that are more suitable for this specific anatomical site and its pathological characteristics.

### 4.5 The necessity of ongoing care after patient discharge

Acute radiation dermatitis is typically defined as skin changes that occur within 90 days after radiotherapy, while changes that occur after more than 90 days are considered chronic radiation dermatitis (52). For patients undergoing three-dimensional intracavitary postoperative radiotherapy, the treatment course lasts about a month, making it highly likely that they may develop radiation dermatitis after discharge. Therefore, ongoing care and continuous monitoring of their skin condition after discharge are especially important. Through telephone follow-up and home care guidance, the effectiveness of clinical care can be extended, and patients can be advised to undergo regular follow-up visits after discharge. This approach helps to effectively reduce the incidence of radiation dermatitis and improves patients’ quality of life and satisfaction (30, 37).

## 5 Conclusions

This study summarizes the evaluation and documentation of radiation dermatitis, health education, general nursing measures, radiation dermatitis management, and continuity care for patients with gynecologic tumor intracavitary postoperative radiotherapy. It provides a basis for developing clinical skin management protocols. However, after conducting the literature search, we found that the relevant articles are primarily concentrated on cervical cancer. This also reflects that the current research focus on radiation dermatitis related to radiotherapy for gynecologic tumors is mainly centered on cervical cancer. The two RCTs we included also fall within the cervical cancer category. Nonetheless, because the mechanisms of radiation dermatitis are similar across different gynecologic tumors—especially since the affected areas are mainly concentrated in the vulva, perineum, and inguinal regions—there is a certain degree of commonality in the available data across different cancer types.

In the future, further randomized controlled trials (RCTs) should be encouraged to investigate radiation dermatitis specifically in endometrial and ovarian cancers, aiming to enhance the evidence for targeted management of radiation dermatitis in these cancer categories.

Additionally, we have summarized the similarities and differences in the occurrence and management of radiation dermatitis during brachytherapy for cervical, ovarian, vaginal, and endometrial cancers in **Table 5**.

**Table 5:** Comparative analysis of radiation dermatitis related to radiotherapy in gynecologic cancers.

Aspect	Shared Features	Differences (By Cancer Type)
Mechanism of Occurrence	Radiation induces DNA damage to skin cells, causing inflammation, erythema, and skin dryness or ulceration	Similar mechanism, but dose intensity vary among cancer types, affecting dermatitis severity
Affected Areas	Usually occurs in the irradiated skin area, primarily in vulvar, perineal, and inguinal regions	- Cervical and vaginal cancers: vulva, vaginal wall, perineal region - Ovarian cancer: less common, mainly due to pelvic irradiation; skin exposure less direct - Endometrial cancer: low incidence, may occur in vagina or perineal area occasionally
Incidence and Severity	Radiation dermatitis is common, ranging from mild erythema to severe ulceration	- Cervical and vaginal cancers: higher incidence - Ovarian and endometrial cancers: less common, usually mild if occurs
Management Principles	Local skin protection, moisturizing, anti-inflammatory treatment, avoiding mechanical irritation, preventing infection	Tailored according to severity, mild cases use moisturizers or topical steroids, severe cases may require dressing changes or antibiotics

Clinicians should determine the recommended levels of evidence based on expert opinions and perform evidence translation according to the FAME (Findings, Appraisal, Management, Evidence) framework. This approach will facilitate the formulation and validation of prevention and management strategies for radiation dermatitis in patients undergoing intracavitary postoperative radiotherapy for gynecologic tumors. Ultimately, it aims to reduce the incidence of radiation dermatitis in these patients, alleviate skin injuries, and improve their quality of life and prognosis.
